# Lysophosphatidylinositol Causes Neurite Retraction via GPR55, G_13_ and RhoA in PC12 Cells

**DOI:** 10.1371/journal.pone.0024284

**Published:** 2011-08-31

**Authors:** Yutaro Obara, Sanae Ueno, Yoshimi Yanagihata, Norimichi Nakahata

**Affiliations:** Department of Cellular Signaling, Graduate School of Pharmaceutical Sciences, Tohoku University, Sendai, Japan; University of Houston, United States of America

## Abstract

GPR55 was recently identified as a putative receptor for certain cannabinoids, and lysophosphatidylinositol (LPI). Recently, the role of cannabinoids as GPR55 agonists has been disputed by a number of reports, in part, because studies investigating GPR55 often utilized overexpression systems, such as the GPR55-overexpressing HEK293 cells, which make it difficult to deduce the physiological role of endogenous GPR55. In the present study, we found that PC12 cells, a neural model cell line, express endogenous GPR55, and by using these cells, we were able to examine the role of endogenous GPR55. Although GPR55 mRNA and protein were expressed in PC12 cells, neither CB_1_ nor CB_2_ mRNA was expressed in these cells. GPR55 was predominantly localized on the plasma membrane in undifferentiated PC12 cells. However, GPR55 was also localized in the growth cones or the ruffled border in differentiated PC12 cells, suggesting a potential role for GPR55 in the regulation of neurite elongation. LPI increased intracellular Ca^2+^ concentration and RhoA activity, and induced ERK1/2 phosphorylation, whereas endogenous and synthetic cannabinoids did not, thereby suggesting that cannabinoids are not GPR55 agonists. LPI also caused neurite retraction in a time-dependent manner accompanied by the loss of neurofilament light chain and redistribution of actin in PC12 cells differentiated by NGF. This LPI-induced neurite retraction was found to be G_q_-independent and G_13_-dependent. Furthermore, inactivation of RhoA function via C3 toxin and GPR55 siRNA knockdown prevented LPI-induced neurite retraction. These results suggest that LPI, and not cannabinoids, causes neurite retraction in differentiated PC12 cells via a GPR55, G_13_ and RhoA signaling pathway.

## Introduction

Cannabinoids, which include the bioactive constituents of the marijuana plant *Cannabis sativa* and its synthetic or endogenous counterparts, modulate a range of central nervous system functions, and affect peripheral sites, such as immune function and the cardiovascular system [1], [2]. Several endogenous cannabinoid ligands have been isolated, including anandamide [3] and 2-arachidonoyl-glycerol (2-AG) [4], [5]. To date, two classical cannabinoid receptors have been identified, specifically cannabinoid receptor type 1 (CB_1_) [6] and cannabinoid receptor type 2 (CB_2_) [7]. CB_1_ is predominantly expressed within the central nervous system [8], whereas CB_2_ is mainly expressed within the immune system [7]. Both cannabinoid receptors are coupled with *Pertussis* toxin-sensitive G_i/o_-proteins [1], and activation of CB_1_ and CB_2_ receptors reduces a forskolin-induced cyclic AMP accumulation [9].

In addition to CB_1_ and CB_2_ receptors, an orphan G-protein-coupled receptor, GPR55, was recently identified as a novel putative cannabinoid receptor [10]. However, GPR55 shares a low homology with the amino acid sequence of CB_1_ (13.5%) or CB_2_ (14.4%). GPR55 was first reported as an orphan receptor expressed extensively in the human brain [11], suggesting that GPR55 regulates neuronal function. Cannabinoids, including Д9-tetrahydrocannabinol (THC), CP55940, anandamide, 2-AG, O1602, and abnormal cannabidiol, are GPR55 agonists, whereas cannabidiol is an antagonist, as determined by GTPγS binding assay [12]. O1602-stimulated GTPγS binding is blocked by Gα_13_ carboxy-terminus and Gα_13_ antibody, suggesting that GPR55 interacts with G_13_. THC increases intracellular Ca^2+^ concentrations ([Ca^2+^]_i_) via GPR55, G_q_ and RhoA, however, some cannabinoids, such as 2-AG and CP55940, have no effect on [Ca^2+^]_i_ [13]. Conversely, anandamide and 2-AG have no effect on GPR55 activation, and CP55940 is a competitive antagonists of GPR55 [14]. Furthermore, cannabinoids, including THC, anandamide, 2-AG, O1602, and abnormal cannabidiol, were shown to have no effect on β-arrestin-dependent ligand-mediated activation of GPR55, and CP55940 was shown to be a GPR55 antagonist/partial agonist [15]. These cannabinoids also do not appear to activate extracellular signal-regulated kinase (ERK) 1/2 via GPR55 [16]. However, it should be mentioned that the majority of the abovementioned studies utilized HEK293 cells that overexpress GPR55. Consequently, there may be inconsistencies in these results, and therefore some of the findings might be controversial [17]. Despite this, it has been demonstrated that lysophosphatidylinositol (LPI) activates ERK1/2 and increases [Ca^2+^]_i_ via GPR55 [16]. There is no evidence that LPI interacts with the other cannabinoid receptors, particularly CB_1_ and CB_2_. Since this study, more detailed signaling pathway and role of GPR55 have been examined using LPI as a GPR55 agonist. For example, LPI promotes RhoA-dependent Ca^2+^ signaling and nuclear factor of activated T cells (NFAT) via GPR55 [14], and inhibits mouse osteoclast formation through the activation of Rho and ERK1/2 [18]. However, the role of GPR55 and LPI in neuronal cells remains unclear.

In the present study, we show that rat PC12 cells, a neuronal model cell line, express endogenous GPR55. Thus, the objective of the present study was to determine the effects of cannabinoids on the signaling and physiological roles of GPR55 in PC12 cells. Herein, we demonstrated that LPI, not cannabinoids, stimulates GPR55 signaling and causes neurite retraction in PC12 cells differentiated by nerve growth factor (NGF).

## Materials and Methods

### Materials

CP55940 and cannabidiol were purchased from Tocris (Ellisville, MO). Anandamide was purchased from Biomol Research Labs, Inc. (Plymouth Meeting, PA). 2-AG was purchased from Cayman Chemical Company (Ann Arbor, MI). TRI Reagent®, LPI, lysophosphatidic acid (LPA), UTP, CGS21680, NGF, luciferin, Hoechst-33258 and anti-neurofilament light chain antibody were purchased from Sigma-Aldrich (St. Louise, MI). Y27632 was purchased from Calbiochem (San Diego, CA). Fura-2 was purchased from Dojindo (Kumamoto, Japan). Phalloidin-Rhodamine was purchased from Molecular Probes (Eugene, OR). Antibodies against phospho-ERK1/2, ERK1/2, glyceraldehyde-3-phosphate dehydrogenase (GAPDH) and horseradish peroxidase (HRP)-conjugated rabbit IgG were from Cell Signaling. Anti-GPR55 was from Enzo Life Sciences (Plymouth Meeting, PA). Anti-RhoA antibody was from Santa Cruz (Santa Cruz, CA). Anti-HA antibody was from Roche (Manheim, Germany). Enhanced chemiluminescence (ECL) assay kit and HRP-conjugated anti-mouse IgG were purchased from GE Healthcare (Buckinghamshire, England). Alexa588-anti-rat IgG antibody and lipofectamine 2000 were purchased from Invitrogen (Grand Island, NY). pSRF/Luciferase was purchased from Promega (Madison, WI). ReverTraAce® was purchased from Toyobo (Osaka, Japan). SYBR® Premix Ex Taq™ (Perfect Real Time) was purchased from Takara (Otsu, Japan). siRNA for rat GPR55 was synthesized by B-Bridge (Mountain View, CA), and the cocktail of three duplexes was used, i.e. 1) sense 5′-GGA GAC AGC UGG AAU ACA UTT-3′ and antisense 5′-AUG UAU UCC AGC UGU CUC CTT-3′; 2) sense 5′-CGA AAG AGA GCC UGC AUC ATT-3′ and antisense 5′-UGA UGC AGG CUC UCU UUC GTT-3′; and 3) sense 5′-GCA GAG UGA AGC AGG GCA UTT-3′ and antisense 5′-AUG CCC UGC UUC ACU CUG CTT-3′. All other chemicals were of reagent grade or the highest quality available. Adenoviruses encoding p115-RGS and C3 were kindly provided by Dr. Hitoshi Kurose (Kyushu University, Fukuoka, Japan), DNA plasmid for HA-GPR55 was kindly provided by Dr. Ken Mackie (Indiana University, Bloomington, IN), GloSensor cAMP and endotoxin-free luciferin were kindly provided by Promega, YM254890 was kindly provided by Astellas Pharma Inc. (Tokyo, Japan), GST-Rhotekin was kindly provided by Dr. Naoki Mochizuki (National Cerebral and Cardiovascular Center Research Institute, Osaka, Japan), and Gα_13_Q266L was kindly provided by Dr. Takeo Saneyoshi (Riken, Wako, Japan).

### Cell culture

PC12 cells were obtained from the Japanese Cancer Research Bank (Tokyo, Japan). The cells were grown in Dulbecco's modified Eagle's medium (DMEM) supplemented with 10% heat-inactivated fetal calf serum (Cell Culture Laboratory, Cleveland, OH), 5% horse serum (Invitrogen), penicillin (50 units/ml), and streptomycin (50 mg/ml) in an incubator containing 5% CO_2_ at 37°C.

### SDS-polyacrylamide gel electrophoresis and Western blotting

Electrophoresis was performed on 8–11% acrylamide gels. Proteins were transferred electrically from the gel onto polyvinylidene difluoride membranes (Millipore, Bedford, MA) via the semi-dry blotting method. Blots were blocked for 1 h with 5% low fat milk in Tris-buffered saline containing 0.1% tween-20 (TBST) at room temperature, and incubated with primary antibodies overnight at 4°C. Blots were washed several times and incubated with HRP-conjugated anti-rabbit or anti–mouse IgG antibody as a secondary antibody in TBST containing 5% low fat milk at room temperature for 2 h. After rinsing with TBST, blots were developed using a chemiluminescence assay kit, and visualized by exposing the chemiluminescence from the membrane to a Hyper-film ECL.

### Assay for neurite outgrowth

Cells were fixed with 4% paraformaldehyde, and the nuclei were stained with Hoechst-33258. Photographs were taken with CELAVIEW-RS100 (Olympus, Tokyo, Japan) [19]. The number of nuclei and total length of neurites were calculated using CELAVIEW software (Olympus, Tokyo, Japan), and then the value of total neurite length divided by the number of nuclei was expressed as a ratio of neurite length per cell (µm/cell). Data were expressed as means ± S.E.M. based on the values of the three wells.

### Luciferase assay

DNA plasmids were transfected into PC12 cells using the transfection reagent Lipofectamine 2000. Briefly, cells were seeded onto 24-well plates at 1×10^5^ (cells/well) and cultivated for a day. DNA plasmids ([0.7–0.8 µg pSRF/luc and 0.2–0.3 µg β-galactosidase] or [(0.45 µg pSRF/luc, 0.1 µg β-galactosidase and 0.45 µg empty vector or G_13_Q266L)]) and transfection reagent (1 µl/tube) were mixed gently in DMEM (10 µl/tube) and incubated for 20 min at room temperature. After addition of DMEM (40 µl/tube), this entire mixture (50 µl/well) was transferred to cultured media, which had been replaced with serum free-DMEM (200 µl). Cells were incubated for 4–6 h at 37°C, and then media was replaced with growth medium (500 µl) containing 10% fetal calf serum and 5% horse serum. For the reporter gene assays, cells were incubated with drugs at 37°C for 6–8 h after serum starvation, and subjected to the luciferase assay. Cells were lysed in lysis buffer (1% Triton X-100, 110 mM K_2_HPO_4_, 15 mM KH_2_PO_4_, pH 7.8) (100 µl/well), and then, after centrifuging lysates to remove cell debris, the supernatant (50 µl/tube) was mixed with 300 µl of assay buffer (25 mM Gly-Gly, 15 mM MgSO_4_, 5 mM ATP, 10 mM NaOH). The luciferase reaction was started by adding 100 µl of luciferin solution (150 µM), and luciferase activity was measured using a luminometer (GENE LIGHT 55, Microtech Nition, Funabashi, Japan). As an internal control, β-actin promoter-driven β-galactosidase activity was measured in the lysates to normalize for transfection efficiency.

### Adenoviral infection

PC12 cells were infected with adenoviruses encoding the RGS domain of p115 Rho GEF and C3 toxin that inactivates G_12/13_ and RhoA, respectively [20], [21]. The GFP gene was introduced to monitor infection efficiency of these G-protein interfering mutants or C3 toxin. The infection was carried out for two days at 100 moi. Control cells were infected with adenovirus encoding GFP. The day after infection, cells were incubated with NGF for a day and stimulated with drugs for indicated times.

### Reverse transcription-polymerase chain reaction (RT-PCR)

Total RNA was isolated from PC12 cells or cerebellar granule cells using the TRI Reagent® according to the manufacturer's protocol. Total RNA was reverse-transcribed using ReverTraAce® and the oligo (dT) primer. Primer sequences used were as follows: CB_1_ receptor sense primer 5′-ATA AGA GGA TCG TCA CCA GG-3′ and antisense primer 5′-AGT TCA GCA GGC AGA GCA TA-3′ (498 bp product); CB_2_ receptor sense primer 5′-AAG CCC TCG TAC CTG TTC AT-3′ and antisense primer 5′-AGG CAC AGC ATG GAG CAG AA-3′ (668 bp product); β-actin sense primer 5′-AGG GAA ATC GTG CGT GAC AT-3′ and antisense primer 5′-TCC TGC TTG CTG ATC CAC AT-3′ (467 bp product); GPR55 sense primer 5′-CTC CCT CCC ATT CAA GAT GA-3′ and antisense primer 5′-AAG ATC TCC AGG GGG AAG AA-3′ (342 bp product), and GAPDH sense primer 5′-ACC ACA GTC CAT GCC ATC AC-3′ and antisense primer 5′-TCC ACC ACC CTG TTG CTG TA-3′ (462 bp product). PCR products were separated by electrophoresis through an agarose gel and stained with ethidium bromide. An image of each gel was digitally captured using FASIII (Toyobo). For quantification of mRNA expression, real-time PCR was carried out in a 20 µl solution containing SYBER Premix Ex Taq (10 µl), RT template (3 µl), water (6 µl) and primers (1 µl) using the DNA engine Opticon System (MJ Research, Waltham, MA). The amount of each PCR product was normalized to GAPDH, and expressed as a percentage change relative to control siRNA treatment.

### Immunostaining

PC12 cells transfected with HA-GPR55 were fixed with 4% paraformaldehyde and stained with anti-HA primary antibody (1∶100 dilution) and Alexa 588-conjugated anti-rat IgG secondary antibody (1∶500 dilution). Then, cells were observed with a fluorescence microscope (Olympus IX70; Olympus, Tokyo, Japan). Also, F-actin protein was stained with Phalloidin-Rhodamine and observed with a confocal laser microscope (DMRB/E, TCS-NT, Leica, Wetzlar, Germany).

### Measurement of [Ca^2+^]_i_

[Ca^2+^]**_i_** levels were measured by monitoring the intensity of fura-2 fluorescence. PC12 cells were washed with modified Tyrode's solution (137 mM NaCl, 2.7 mM KCl, 1.0 mM MgCl_2_, 0.18 mM CaCl_2_, 10 mM HEPES, 5.6 mM glucose, pH 7.4). Cells were then loaded with 1 µM fura-2/AM for 30 min at 37°C. Fluorescence intensity of fura-2 (excitation wavelength at 340 nm and 380 nm, and emission wavelength at 510 nm) was measured using a spectrofluorometer (FP-6500, JASCO, Tokyo, Japan).

### Measurements of cAMP levels

The GloSensor cAMP, a fusion gene of the cAMP-binding domain of protein kinase A and firefly luciferase, was utilized to measure cAMP levels in living cells. By binding to cAMP, the conformation change results in increased luciferase activity [22], [23], [24], [25]. Briefly, cells were transfected with GloSensor cAMP and β-galactosidase, and were incubated in Tyrode's solution (NaCl 137 mM, KCl 2.7 mM, MgCl_2_ 1.0 mM, CaCl_2_ 1.8 mM, NaH_2_PO_4_, 0.4 mM, Glucose 5.6 mM, Hepes 10 mM, pH 7.4) containing D-luciferin (2 mM) (100 µl/well) for two hours at room temperature. Then, cells were stimulated with 10× drugs (11 µl/well), and luminescence was measured with a luminometer (GloMax, Promega, Madison, IL). As an internal control, β-actin promoter-driven β-galactosidase activity was measured in lysates to normalize for transfection efficiency.

### Affinity assay for RhoA activation

The GST fusion protein of the Rho-binding domain of Rhotekin was expressed in *Escherichia coli* following induction with isopropyl-β-D-1-thio-galactopyranoside. Cells were lysed in ice-cold lysis buffer (50 mM Tris-HCl (pH 8.0), 10% glycerol, 1% nonidet P-40, 200 mM NaCl, 2.5 mM MgCl_2_, 1 mM phenylmethylsulfonyl fluoride, 1 µM leupeptin, 10 µg/ml soybean trypsin inhibitor, 10 mM NaF, 0.1 µM aprotinin, and 1 mM NaVO_4_). Protein concentrations were quantified using the Bradford protein assay, and active RhoA was isolated, as described previously [26]. Equivalent amounts of supernatant (1000 µg total protein) was incubated with GST-Rhotekin (including the RhoA binding domain) coupled to glutathione beads. Following an hour of incubation at 4°C, beads were pelleted and rinsed three times with ice-cold lysis buffer, and proteins were eluted from the beads using 2× Laemmli buffer.

### Statistical analysis

Data are expressed as means ± S.E.M. Significant differences were determined using Student's *t*-test, Dunnett's or Tukey-Kramer's multiple comparison tests.

## Results

### Expression and intracellular localization of GPR55 in PC12 cells

**Figure 1:**
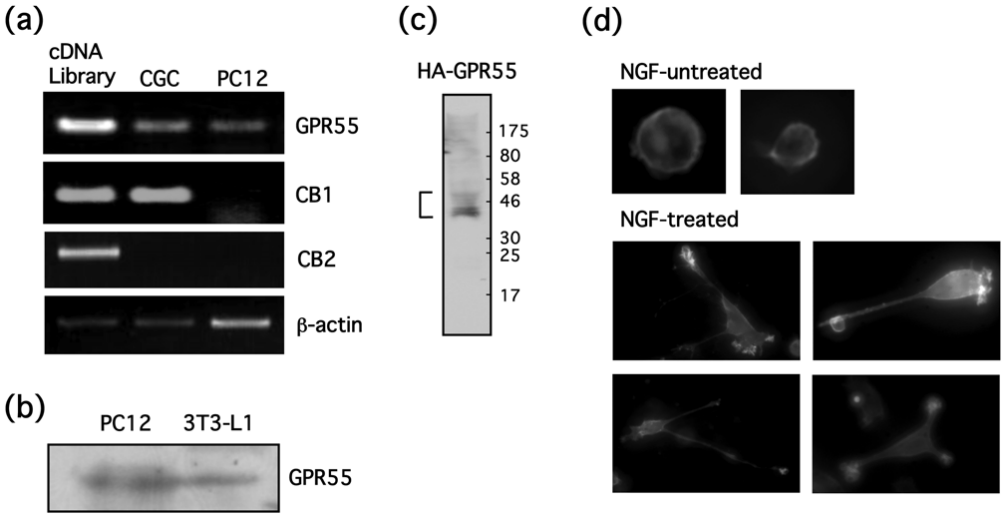
PC12 cells express endogenous GPR55. (**a**) Total RNA was extracted from PC12 cells or rat cerebellar granule cells (CGC), and RT-PCR was performed using a primer mixture corresponding to rat GPR55, CB_1_ or CB_2_. β-Actin was used as a control gene. (**b**) GPR55 protein expression in PC12 cells and mouse 3T3-L1 adipocytes was determined by Western blotting. (**c**) PC12 cells were transfected with HA-GPR55, and protein expression was determined by Western blotting. (**c**) PC12 cells were transfected with HA-GPR55 and incubated in the presence or absence of NGF (100 ng/ml) for a day. Then, immunostaining with HA antibody was performed and localization of GPR55 was observed under a fluorescence microscope.

Gene expression of cannabinoid-related receptors in PC12 cells was determined by RT-PCR (Figure 1a). PC12 cells express GPR55 mRNA, but not CB_1_ or CB_2_ mRNA, whereas rat cerebellar granule neurons express both GPR55 and CB_1_ as we recently reported [27]. PC12 cells also expressed GPR55 protein as mouse 3T3-L1 adipocytes (Figure 1b). Intracellular localization of GPR55 in PC12 cells was determined. HA-GPR55, where the major band was about 37 kD, and the minor bands, which were assumed to be glycosylated, were also visualized (Figure 1c). HA-GPR55 was predominantly localized on plasma membranes in undifferentiated PC12 cells (Figure 1d). However, in PC12 cells that are differentiated by NGF, HA-GPR55 was abundantly localized at the tip of neurites or on the ruffled border, in addition to the plasma membrane, where neurite extension is regulated by small G-proteins, including Rho, Rac1 and Cdc42 [28] (Figure 1d), thus localization of GPR55 may suggests a potential role for this protein in the regulation of neurites.

### LPI causes G_q_-mediated Ca^2+^ increase and ERK1/2 phosphorylation and G_13_-mediated Rho activation

**Figure 2:**
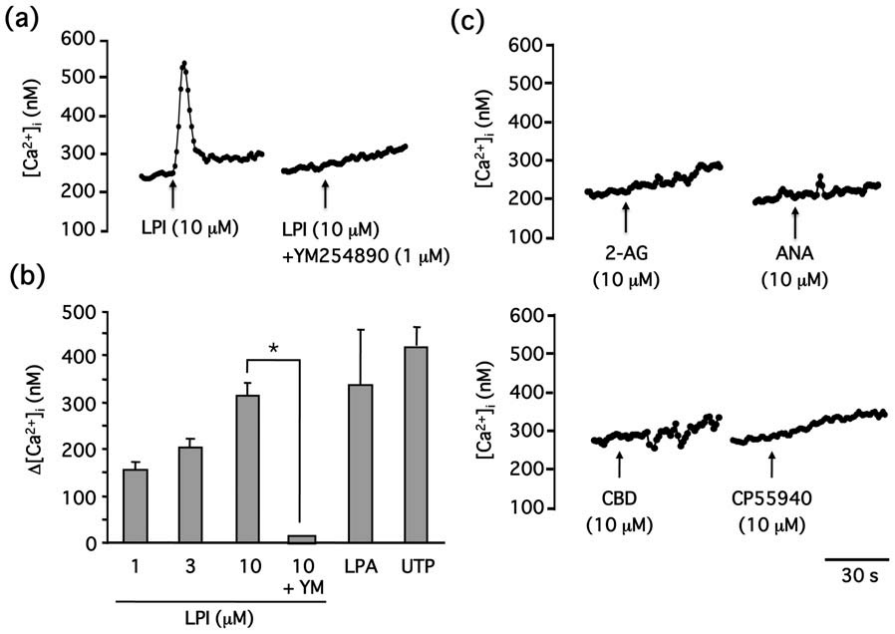
LPI, not cannabinoids, increase [Ca^2+^]_i_ levels in a G_q_-dependent manner in PC12 cells. (**a**) PC12 cells loaded with fura-2 were stimulated with LPI (10 µM) in the presence or absence of YM254890 (1 µM), and [Ca^2+^]_i_ levels were determined. (**b**) PC12 cells loaded with fura-2 were stimulated with LPI (1, 3 or 10 µM), LPA (10 µM) or UTP (100 µM) in the presence or absence of YM254890 (1 µM), and [Ca^2+^]_i_ levels were determined. Data are represented by means ± S.E.M. (n = 3). YM254890 significantly blocked the increase in [Ca^2+^]_i_ by LPI (*P<0.05). (**c**) PC12 cells loaded with fura-2 were stimulated with cannabinoids, including 2-arachidonoylglycerol (2-AG, 10 µM), anandamide (ANA, 10 µM), cannabidiol (CBD, 10 µM) and CP55940 (10 µM), and [Ca^2+^]_i_ levels were determined.

**Figure 3:**
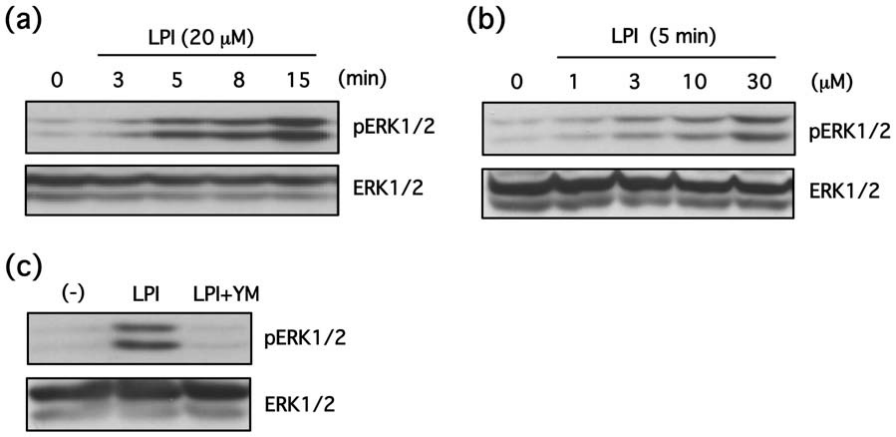
LPI induces phosphorylation of ERK1/2 in a time and concentration-dependent manner in PC12 cells. (**a**) PC12 cells were stimulated with LPI (20 µM) for indicated periods, and then phosphorylation of ERK1/2 was determined by Western blotting. (**b**) PC12 cells were stimulated with LPI (1–30 µM) for 5 min, and phosphorylation of ERK1/2 was examined by Western blotting. (**c**) PC12 cells were stimulated with LPI (20 µM) for 5 min in the presence or absence of YM254890 (1 µM), and then phosphorylation of ERK1/2 was determined by Western blotting.

Although LPI is widely recognized as a GPR55 agonist [16], [29], the role of cannabinoids in GPR55 regulation remains unclear. Given that most of these studies were conducted in HEK293 cells that overexpress GPR55, we used PC12 cells that express endogenous levels of GPR55 to assess the role of LPI and cannabinoids in GPR55 activation by measuring [Ca^2+^]_i_. LPI induced a transient and concentration-dependent increase in [Ca^2+^]_i_ that was completely blocked by the G_q_ inhibitor, YM254890 (1 µM) [30] (Figures 2a and 2b). Additionally, the potency of LPI (10 µM) was comparable to that of LPA and UTP. However, cannabinoids, including 2-AG (10 µM), anandamide (10 µM), cannabidiol (10 µM) and CP55940 (10 µM), did not affect [Ca^2+^]_i_ (Figure 2c). In addition to Ca^2+^ release, ERK1/2 phosphorylation was investigated. LPI also induced ERK1/2 phosphorylation in a time and concentration-dependent manner (Figures 3a and 3b). This ERK1/2 phosphorylation was also completely blocked by YM254890, as observed in Figure 2a (Figure 3c), suggesting that ERK1/2 phosphorylation may be G_q_-dependent.

**Figure 4:**
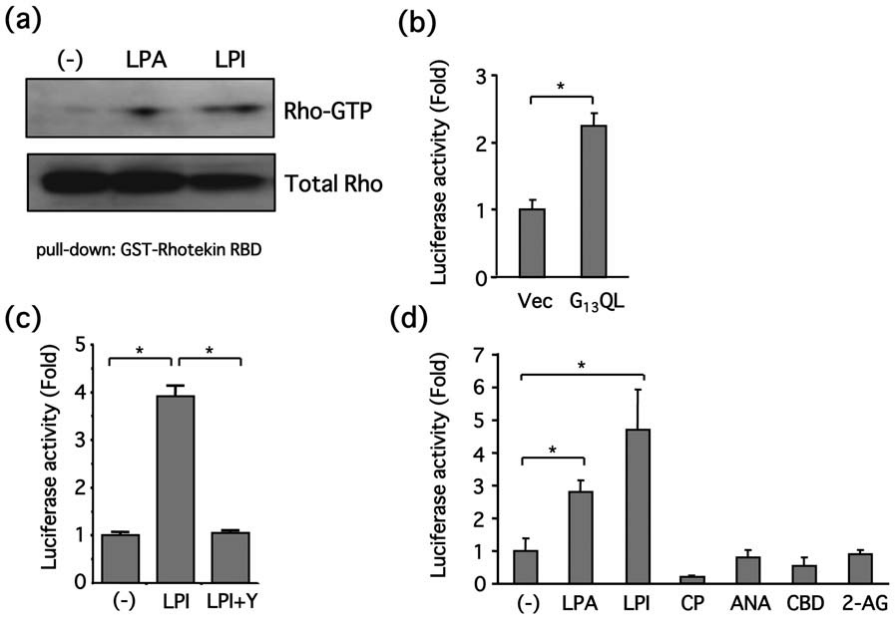
LPI, not cannabinoids, activates RhoA in PC12 cells. (**a**) PC12 cells were stimulated with LPA (10 µM) or LPI (10 µM) for 5 min, and then Rho-GTP was precipitated with GST-Rhotekin RBD, followed by Western blotting. (**b**) PC12 cells were co-transfected with pSRF/luc and Gα_13_Q266L or an empty vector, and then luciferase activity was measured as an index of RhoA activation. Data represent means ± S.E.M. (n = 3). Gα_13_Q266L significantly increased RhoA activity (*P<0.05). (**c**) PC12 cells were transfected with pSRF/luc, and then stimulated with LPI (10 µM) for 6 h in the presence or absence of Y27632 (Y, 10 µM). Luciferase activity was measured as an index of RhoA activation. Data represent means ± S.E.M. (n = 3). LPI significantly increased RhoA activity, which was reversed by Y27632 (*P<0.05). (**d**) PC12 cells were transfected with pSRF/luc, and then stimulated with LPA (10 µM), LPI (10 µM), CP55940 (10 µM), anandamide (ANA, 10 µM), cannabidiol (CBD, 10 µM) or 2-arachidonoylglycerol (2-AG, 10 µM) for 6 h. Luciferase activity was measured as an index of RhoA activation. Data are represented by means ± S.E.M. (n = 3). Both LPA and LPI significantly increased RhoA activity, whereas RhoA activity was not affected by any of the cannabinoids tested (*P<0.05).

RhoA was activity also examined through the pull-down assay with the GST-Rhotekin Rho-binding domain as an index of GPR55 interaction with LPI or cannabinoids. LPI (10 µM, 5 min) activated RhoA similarly to LPA (10 µM, 5 min) (Figure 4a). RhoA-dependent serum-response element (SRE) activation was also measured. Overexpression of the GTPase-deficient Gα_13_ mutant (G_13_QL) resulted in significant SRE-activation (Figure 4b). Additionally, LPI (10 µM, 6 h) increased SRE activity, which was completely reversed by a Rho-associated coiled-coil containing protein kinase (ROCK) inhibitor, Y27632 (10 µM) (Figure 4c). However, cannabinoids, including CP55940 (10 µM), anandamide (10 µM), cannabidiol (10 µM) and 2-AG (10 µM), did not increase SRE activity (Figure 4d). These findings suggest that LPI, and not the cannabinoids tested in the present study, can activate RhoA in PC12 cells.

It has previously been demonstrated that GPR55 can couple with G_q_ and G_13_, and as a result increase [Ca^2+^]_i_ and RhoA activity [12], [13]. However, the effects of GPR55 on cAMP levels have not yet been demonstrated. Thus, in the present study, we measured intracellular cAMP levels in living cells using a novel fusion gene of the cAMP-binding domain of protein kinase A and firefly luciferase [22], [23], [24], [25]. This cAMP biosensor responds to cAMP, and the luminescence levels increase based on intracellular cAMP concentrations. An adenosine A_2_ receptor agonist, CGS21680 (10 µM), increased intracellular cAMP levels. However, LPI (10 µM) and the abovementioned cannabinoids (10 µM) did not result in any cAMP production, despite GPR55 overexpression (Figure S1), suggesting that GPR55 does not interact with Gα_s_.

### LPI causes neurite retraction via GPR55, G_13_ and RhoA

**Figure 5:**
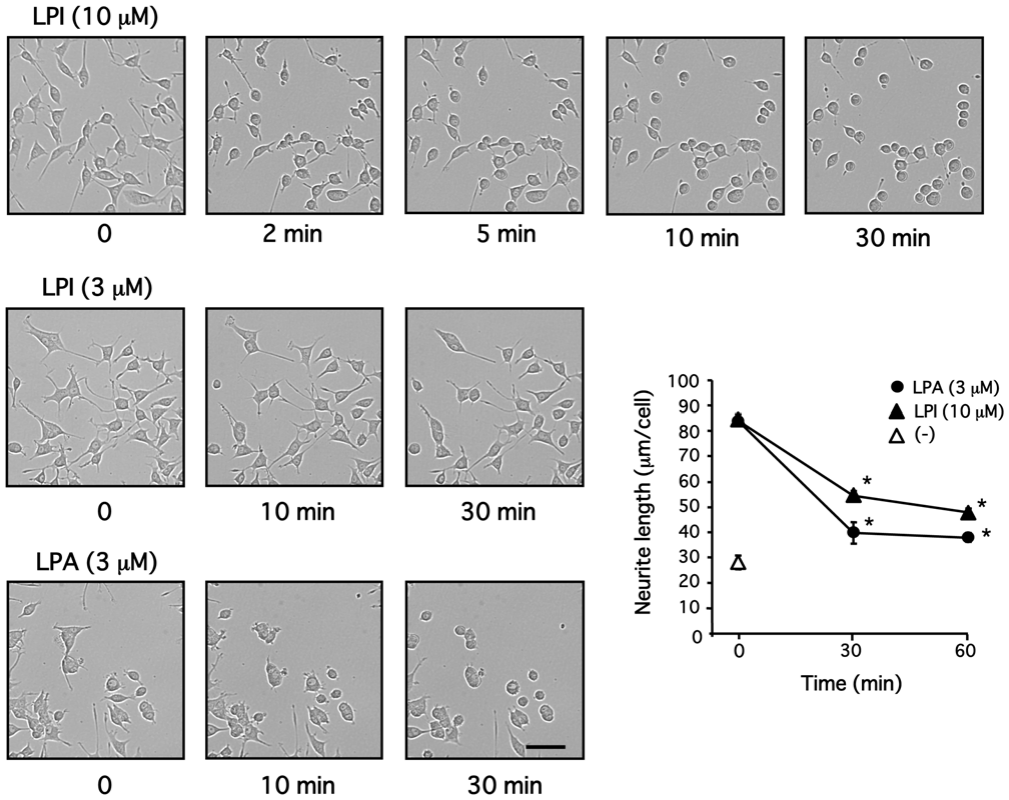
LPI induces neurite retraction in PC12 cells differentiated by NGF. PC12 cells were differentiated by NGF (100 ng/ml) for a day, and then stimulated with LPI (3 or 10 µM) or LPA (3 µM) for indicated periods. Morphology of PC12 cells was observed and neurite length was measured, as described in *Materials and Methods*. Scale bar = 50 µm. Data are represented by means ± S.E.M. (n = 3). LPI (10 µM) or LPA (3 µM) significantly caused neurite retraction (*P<0.05).

**Figure 6:**
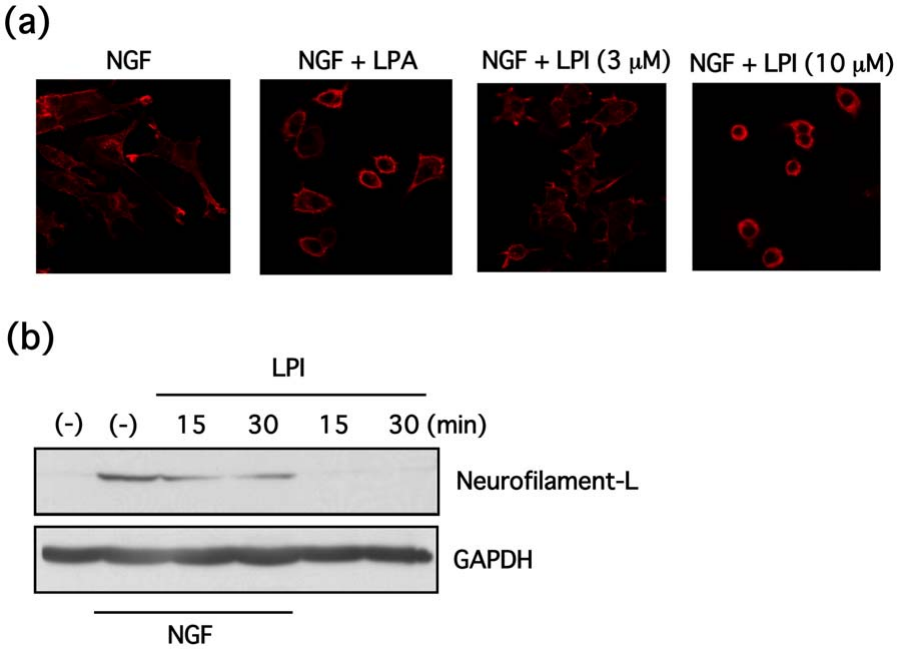
LPI induces neurite retraction accompanied by redistribution of β-actin and loss of neurofilament light chain in PC12 cells. (**a**) PC12 cells were differentiated by NGF (100 ng/ml) for a day, and then stimulated with LPI (3 or 10 µM) or LPA (3 µM) for 15 min. Cells were fixed and stained with rhodamine-phalloidin (5 U/ml), and observed with a confocal laser microscope. (**b**) PC12 cells were incubated in the presence or absence of NGF (100 ng/ml) for a day, and then stimulated with LPI (10 µM) for 15 or 30 min. Contents of neurofilament light chain and GAPDH were determined by Western blotting.

Further, the effects of LPI on neurite elongation or retraction were examined. In undifferentiated PC12 cells, LPI showed no effect on neurite outgrowth (data not shown). However, in PC12 cells differentiated by NGF (100 ng/ml, 24 h), LPI (10 µM) and LPA (3 µM), but not LPI (3 µM), caused rapid neurite retraction (Figure 5). This dramatic change in neurite shape, induced by LPI, was accompanied by redistribution of F-actin and loss of the neurofilament light chain (Figure 6).

**Figure 7:**
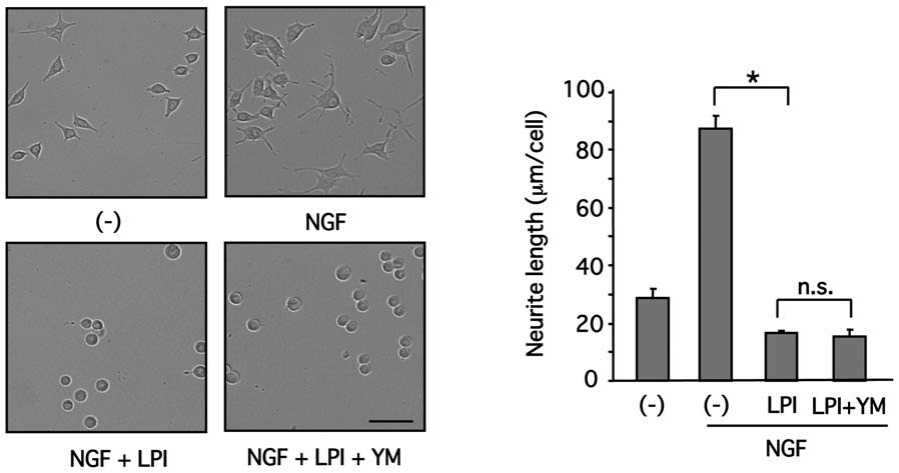
G_q_ is not involved in LPI-induced neurite retraction in PC12 cells differentiated by NGF. PC12 cells were differentiated by NGF (100 ng/ml) for a day, and then stimulated with LPI (10 µM) for 30 min in the presence or absence of YM254890 (1 µM). Morphology of PC12 cells was examined and neurite length was measured, as described in *Materials and Methods*. Scale bar = 50 µm. Data are represented by means ± S.E.M. (n = 3). LPI (10 µM) significantly induced neurite retraction (*P<0.05), however YM254890 did not reverse the effect.

**Figure 8:**
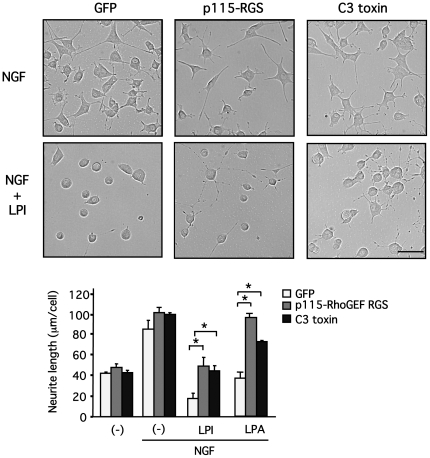
G_13_ and RhoA are involved in LPI-induced neurite retraction in PC12 cells differentiated by NGF. PC12 cells were infected with adenoviruses encoding p115-RGS or C3 toxin (100 moi) and differentiated by NGF (100 ng/ml) for a day. Cells were stimulated with LPI (10 µM) or LPA (3 µM) for 30 min. Morphology of PC12 cells was examined and neurite length was measured, as described in *Materials and Methods*. Scale bar = 50 µm. Data are represented by means ± S.E.M. (n = 3). p115-RGS and C3 significantly reversed LPI (10 µM) or LPA (3 µM)-induced neurite retraction (*P<0.05).

To determine the signaling pathway responsible for the LPI-induced neurite retraction, cells differentiated by NGF were pretreated with YM254890, and then stimulated with LPI. Neurite retraction induced by LPI (10 µM) was not affected by YM254890 (1 µM) (Figure 7). Cells were infected with adenoviruses encoding RGS domain of p115 RhoGEF (p115-RGS) or C3 toxin [20], [21] to determine the involvement of G_13_. p115-RGS binds to the βγ subunit-dissociated Gα_12_ or Gα_13_ and promotes GTP hydrolysis by activating GTPase activity. C3 toxin causes ADP ribosylation to Rho, one of the major effectors of Gα_12_ or Gα_13_. Cells were also infected with adenoviruses encoding for GFP alone, and these cells served as a control. LPI (10 µM) reduced neurite length, and this effect was significantly reversed through inhibition of Gα_13_ or RhoA function (Figure 8). Similarly, LPA-treated cells (3 µM) also displayed neurite retraction via activation of these G-proteins, and served as a positive control. These findings suggest that LPI-induced neurite retraction is G_q_-independent, and Gα_13_ and RhoA-dependent.

**Figure 9:**
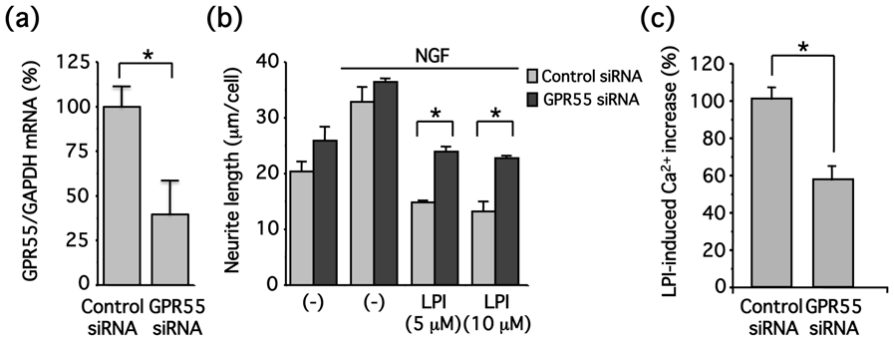
GPR55 is involved in LPI-induced neurite retraction in PC12 cells differentiated by NGF. (**a**) PC12 cells were transfected with GPR55 siRNA. Two days after transfection, total RNA was extracted and real-time RT-PCR was performed using primers corresponding to rat GPR55 or GAPDH. (**b**) PC12 cells were transfected with GPR55 siRNA, and then cells were differentiated by NGF (100 ng/ml) for a day. Cells were stimulated with LPI (5 or 10 µM) for 30 min. Morphology of PC12 cells was examined and neurite length was measured, as described in *Materials and Methods*. Data are represented by means ± S.E.M. (n = 3). GPR55 siRNA significantly reversed LPI (5 or 10 µM)-induced neurite retraction (*P<0.05). (**c**) PC12 cells were transfected with GPR55 siRNA, and then cells loaded with fura-2 were stimulated with LPI (10 µM), and [Ca^2+^]_i_ levels were determined.

Lastly, we attempted to determine whether the effects of LPI on neurite retraction were via GPR55. It was previously demonstrated that cannabidiol is a GPR55 antagonist [12]. However, in our study, cannabidiol did not affect LPI-induced increases in [Ca^2+^]_i_ (data not shown). In addition to cannabidiol, it has been reported that CP55940 is a competitive GPR55 antagonist [14] although other studies demonstrated CP55940 showed a GPR55 agonistic activity or no effect [12], [16]. However, neither cannabidiol nor CP55940 blocked LPI-induced neurite retraction in this study (data not shown), thereby suggesting that cannabidiol and CP55940 are not actually GPR55 antagonists. Thus, we used an siRNA approach to investigate the involvement of GPR55. GPR55 mRNA levels in cells treated with GPR55 siRNA were decreased by 61% (Figure 9a), and LPI-induced neurite retraction was significantly reversed in these GPR55 siRNA-treated cells (Figure 9b) suggesting that LPI promotes neurite retraction via GPR55. LPI-induced [Ca^2+^]_i_ elevation via G_q_ was also significantly blocked by GPR55 knock down (Fig. 9c) in addition to G_13_ and RhoA-dependent neurite retraction.

## Discussion

**Figure 10:**
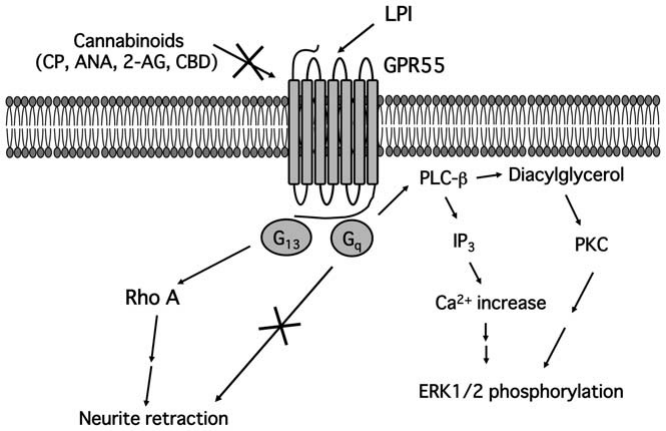
Putative GPR55 signaling in PC12 cells.

In the present study, we have demonstrated that LPI promotes neurite retraction in PC12 cells through GPR55, G_13_ and Rho, whereas cannabinoids, including anandamide, 2-AG, CP55940 and cannabidiol, do not interact with GPR55 in these cells (Figure 10).

GPR55 was initially indentified as a novel target of cannabinoids, where it was demonstrated that endogenous, synthetic and plant-derived cannabinoids, including THC, CP55940, anandamide, 2-AG, O1602, and abnormal cannabidiol are GPR55 agonists, while cannabidiol is an antagonist determined by GTPγS binding assay [12]. Since this initial discovery, many conflicting observations have been made suggesting that cannabinoids do not affect GPR55 signaling [17]. Nevertheless most of these studies utilized HEK293 cells that overexpress GPR55, there results are inconsistent and remain controversial with no clear explanation for this discrepancy [17]. Therefore, in the present study, we attempted to examine the agonistic effects of cannabinoids on endogenous GPR55. We did not observe any agonistic effects of cannabinoids on GPR55, as demonstrated by no changes in [Ca^2+^]_i_ and Rho activity levels (Figures 2 and 4). In fact, we found that only LPI activated GPR55 signaling in PC12 cells. Unfortunately, the explanation for why cannabinoids do not exhibit agonistic effects on GPR55 in PC12 cells remains unknown.

GPR55 mRNA is expressed in the human brain determined by northern blotting [11]. Recently, an intracellular phospholipase A_1_, DDHD1, and cytosolic phospholipase A_2_, were identified as LPI-synthesizing enzymes [31], [32]. DDHD1 is widely distributed and highly expressed in the brain [33], and therefore, considerable levels of LPI may be synthesized in the brain. In fact, the rat brain contains 37.5 nmol/g tissue of LPI [29]. Thus, we hypothesize that endogenous GPR55, whose function is unclear, plays an important role in the nervous system.

In differentiated PC12 cells, GPR55 is abundantly localized at tips of neurites and on membranes of the ruffled border, in addition to plasma membranes, where neurite extension is regulated by small G-proteins, such as Rho, Rac1 and Cdc42 [28] (Figure 1d), suggesting that GPR55 may be involved in the regulation of neurites. Although GPR55 stimulation with LPI did not promote neurite outgrowth, LPI did trigger dynamic neurite retraction with a loss of neurofilament light chain and actin rearrangement within 30 minutes (Figures 5 and 6). Furthermore, the LPI-induced neurite retraction appeared to be Gα_13_ and Rho-dependent, and Gα_q_-independent (Figures 7 and 8). With respect to the roles of heterotrimeric and small G-proteins in neurite retraction, it has been shown that GPCR agonists that activate Gα_12_, Gα_13_ or small G-protein, Rho, can cause similar neurite retraction in primary cultured hippocampal neurons, PC12 cells or N1E-115 neuroblastoma cells. For example, overexpression of constitutively active mutants of Gα_12_, Gα_13_ or Rho results in growth cone collapse and axonal retraction in hippocampal neurons. A similar effect occurs with thrombin and LPA, which activate the Gα_12_/_13_-Rho signaling pathway [34]. Another study demonstrated that LPA induces growth cone collapse, neurite retraction and cell flattening in differentiated PC12 cells, whereas C3 toxin-treated neurites are resistant to retraction by LPA [35]. Imaging analysis in N1E-115 neuroblastoma cells revealed that RhoA activity in shaft leads to neurite retraction and that in peripheral domain of growth cones contributes to stabilization of growth cone [36]. These reports support our hypothesis that LPI induces neurite retraction via the GPR55/Gα_13_/Rho signaling pathway. In the present study, LPI also stimulated phosphorylation of ERK1/2 through G_q_ in PC12 cells (Figure 3). It is generally believed that sustained activation of ERK1/2 is necessary and sufficient for neurite outgrowth in PC12 cells, which is achieved through the activation of transcription factors that enhance gene expression for neural differentiation. In fact, pharmacological inhibition of ERK1/2 (i.e. using U0126 and PD98059) suppresses neurite outgrowth [19], [26], whereas the constitutively active mutant of MEK induces neurite outgrowth in PC12 cells [37]. In the present study, it was found that LPI-induced neurite retraction occurred in conjunction with ERK1/2 phosphorylation. Therefore, it is suggested that RhoA activity may be dominate the effects of ERK1/2 signaling, and thus, cells cannot extend their neurites while RhoA is activated. In fact, NGF promotes neurite outgrowth in PC12 cells while ERK1/2 is activated and RhoA is inhibited [38].

LPI is synthesized by phospholipase A-mediated removal of one of the acyl moieties of phosphatidylinositol. A commercially available LPI reagent, used in the present study, is prepared from soybean phosphatidylinositol hydrolyzed by phospholipase A_2_. The main fatty acid group is palmitic acid esterified at *sn*-1 position. Recently, it was shown that 2-arachidonoyl LPI (with arachidonic acid at *sn*-2) is a more potent ligand for GPR55 than other LPI molecules, and the most predominant fatty acyl moiety is stearic acid (50.5%) followed by arachidonic acid (22.1%) in the brain [29]. Therefore, 2-arachidonoyl LPI is a better candidate for a physiological GPR55 ligand. Future studies that examine the potency of various LPI molecules on GPR55 are necessary.

Phospholipase A_1/2_ is activated in inflammatory responses, during which lysophospholipids, including LPI, are generated with major inflammatory mediators such as arachidonic acids. Therefore, it can be speculated that LPI may play an important role in inflammatory neurodegenerative diseases such as Alzheimer's disease, Parkinson's disease, amyotrophic lateral sclerosis and multiple sclerosis. Whereas certain cannabinoid-related compounds have been suggested to have promising effects in such diseases [9], LPI/GPR55 signaling may actually enhance the symptoms of these diseases because of LPI-induced loss of neural cell function. Furthermore, it has previously been shown that bee venom-mediated stimulation of phospholipase A_2_ and generation of LPI promote secretion of insulin from pancreatic islet cells, however involvement of GPR55 in this process was not established [39]. Despite this, these findings suggest that LPI/GPR55 regulate insulin secretion, and that drugs that target GPR55 may be used in the treatment of diabetes. Additionally, LPI stimulates catecholamine secretion in PC12 cells [40], although this study did not examine the involvement of GPR55. Furthermore, these findings are corroborated by our preliminary experiments, where we also observed that LPI induced marginal catecholamine secretion (Obara et al., unpublished observation). Since lysophospholipids have detergent-like properties and affect the functions of ion channels and receptors on plasma membranes, GPR55 involvement requires careful examination.

In conclusion, we demonstrated that LPI/GPR55 signaling results in neurite retraction via G_13_ and RhoA in PC12 cells, and that cannabinoids did not exhibit a similar effect on GPR55 signaling as LPI. Development of GPR55-specific agonists or antagonists may have a therapeutic potential in the treatment of inflammatory neurodegenerative diseases. Furthermore, studies using animal models, such as GPR55 knockout mice, to examine the physiological role of GPR55 *in vivo* are essential.

## Supporting Information

Figure S1 **LPI and cannabinoids do not increase intracellular cAMP levels in PC12 cells.** PC12 cells were co-transfected with a cAMP indicator (i.e. a fusion gene of cAMP-binding domain of protein kinase A and firefly luciferase), as well as HA-GPR55 or empty vector. Then, cells were stimulated with LPI (10 µM), anandamide (ANA, 10 µM), 2-arachidonoylglycerol (2-AG, 10 µM), CP55940 (10 µM) or CGS21680 (10 µM) for 15 min, and luciferase activity was measured as an index of intracellular cAMP levels in living cells as described in *Materials and Methods*. Data are represented by means ± S.E.M. (n = 3). CGS21680 significantly increased cAMP levels, whereas cannabinoids and LPI did not.
(TIF)
