# Supplementary figures and images for: Lysophosphatidylinositol Causes Neurite Retraction via GPR55, G13 and RhoA in PC12 Cells
Source: PLoS One. 2011 Aug 31;6(8):e24284. doi: 10.1371/journal.pone.0024284 (PMC3164175; doi:10.1371/journal.pone.0024284)

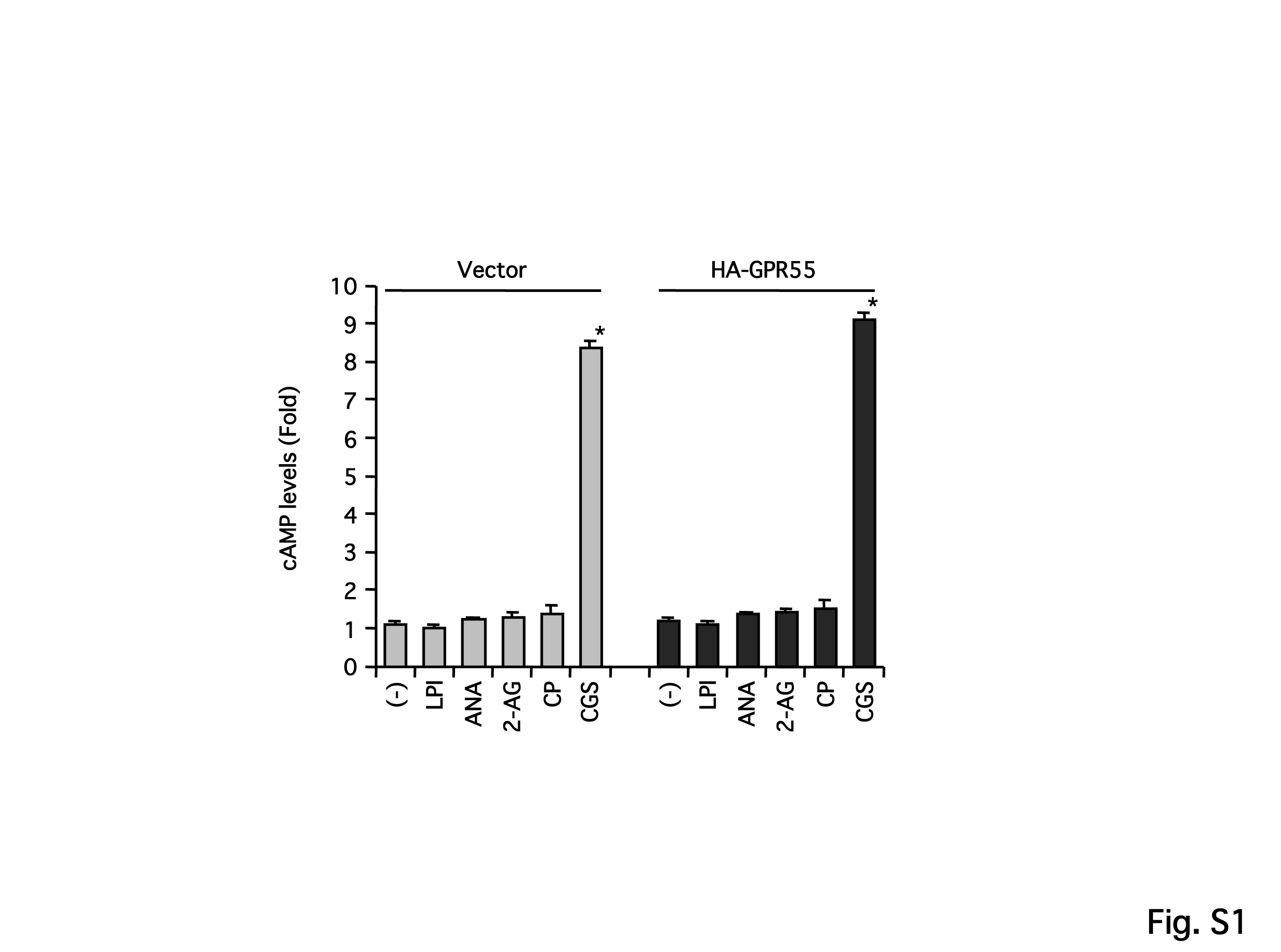

Supplement: Figure S1 — LPI and cannabinoids do not increase intracellular cAMP levels in PC12 cells. PC12 cells were co-transfected with a cAMP indicator (i.e. a fusion gene of cAMP-binding domain of protein kinase A and firefly luciferase), as well as HA-GPR55 or empty vector. Then, cells were stimulated with LPI (10 µM), anandamide (ANA, 10 µM), 2-arachidonoylglycerol (2-AG, 10 µM), CP55940 (10 µM) or CGS21680 (10 µM) for 15 min, and luciferase activity was measured as an index of intracellular cAMP levels in living cells as described in Materials and Methods . Data are represented by means ± S.E.M. (n = 3). CGS21680 significantly increased cAMP levels, whereas cannabinoids and LPI did not. (TIF) [file pone.0024284.s001.tif]
